# Which sites better represent the sensory function of hands in convalescent stroke patients? A study based on electrophysiological examination

**DOI:** 10.3389/fnins.2022.1065629

**Published:** 2023-01-11

**Authors:** Yu Liu, Jiang Ma, Hong Li, Wan-ying Shi, Zheng-hua Xiao, Qian Yang, Qing-qing Zhao, Fang Wang, Xiao-lin Tao, Yun-fei Bai

**Affiliations:** ^1^Department of Rehabilitation Medicine, Shijiazhuang People’s Hospital, Shijiazhuang, China; ^2^Physical Education College, Hebei Normal University, Shijiazhuang, China; ^3^Department of Electrophysiology, Shijiazhuang People’s Hospital, Shijiazhuang, Hebei, China; ^4^School of Nursing and Rehabilitation, North China University of Science and Technology, Tangshan, Hebei, China

**Keywords:** stroke, sensory, SEP, light touch sensation, 2-PD

## Abstract

**Background:**

Assessing hand sensation in stroke patients is necessary; however, current clinical assessments are time-consuming and inaccurate.

**Objective:**

This study aimed to explore the nature of light touch sensation and two-point discrimination (2-PD) of different hand sites in convalescent stroke patients based on somatosensory evoked potentials (SEP).

**Methods:**

Light touch sensation and 2-PD of the thumb, the index finger, the little finger, thenar, and hypothenar were measured (*n* = 112) using sensory measurement tools. Sensory differences among the hand sites were then compared. The correlation analysis between SEP and the hemiplegic hand function was made. Sensory functions were divided into three levels: sensory intactness, sensory impairment, and sensory loss.

**Results:**

Light touch sensations were mainly associated with sensory impairment in the finger and palm region. The 2-PD of the finger region was mainly sensory loss and that of the palm region was mainly sensory impairment. There was no statistical difference in the light touch sensation among the sites of the hand. The correlation coefficients between the 2-PD and SEP N20 amplitudes differed. The correlation coefficients of the thenar and hypothenar were the smallest, and that of the finger was the largest. Light touch sensation and 2-PD in patients with stroke were related to the hemiplegic hand function.

**Conclusion:**

Any site on the hand could be selected as the measurement site for light touch sensation. The little finger and hypothenar may be appropriate sites when screening for 2-PD. To improve the patient’s recovery they could receive more sensory stimulation of the hand.

## 1. Introduction

Nearly 85% of patients with stroke reportedly have sensory dysfunction (Wang et al., 2017; GBD 2017 Mortality Collaborators, 2018). Sensory dysfunction can affect the motor function, activities of daily living, and social participation of patients with stroke (Kessner et al., 2016; Chen et al., 2018). Increasing clinical attention has been paid to the recovery of motor function in patients with stroke, and less attention has been given to the recovery of sensory function. The reason for this may be that motor dysfunction is more easily observed than sensory dysfunction, and the impact of sensory dysfunction on patients’ activities of daily living and participation is often overlooked when motor impairment is more severe. Doctors perform sensory assessments cursorily and do not have a full picture of how the patient is feeling. As motor function improves, sensory problems become more pronounced, which affects the patient’s fine movement and motor control (Carey et al., 2018). Sensory assessment scales, which are less commonly used, do not have sufficient reliability and require a certain level of cognitive ability. Physical examinations provide a quick overview of the patient’s overall sensory function but lack objectivity. Therefore, a comprehensive, objective, and clinically efficient assessment method is needed. In addition, there is evidence that sensory retraining helps the recovery of sensory and modulated sensorimotor integration in convalescent stroke patients and improves activities of daily living and motor function in the upper limbs and hands, with different sensory stimulation sites have different effects (Diego et al., 2013; Brown et al., 2018; Lim, 2019). Therefore, selecting the right area for sensory stimulation is important for improving the efficiency of clinical rehabilitation.

The upper limbs and hands play a key role in precise gripping, motor control, and the manipulation of objects (Tonak et al., 2021) and have a significant impact on patients’ activities of daily living (Tashiro et al., 2021). The upper limb and palm of the hand have high sensory thresholds. Most sensory receptors located in the fingers and palm play different processing roles in sensory coding. Merkel cells, mainly located in the tips of the thumb, the index and middle fingers, and thenar, perceive the material of objects in contact with the skin, and Merkel cells contain the receptors that are important for monofilament touch and two-point discrimination (2-PD) (Feng et al., 2018). The large pachytene microsomal receptor area senses which part of the hand is being stimulated and then localizes the sensation (Germann et al., 2020). Normal touch in mammals requires the function of brain sodium channels in receptors, which may have implications for touch and potentially compound sensory functions when damaged (Price et al., 2000). There was no significant difference in light touch sensation in the two sides of the forearms of patients with stroke, and sensory dysfunction sites were mainly in the fingers (Peng et al., 2018). Stroke lesions are located in the central nervous system, and measurement points are generally not selected according to the distribution of the peripheral nervous system. Current measurement points for sensory assessment are mainly selected in the areas of the hand and upper limb where the function is achieved, such as the thumb and the index finger. Patients with stroke have been shown to have different sensory thresholds among the five fingers on the affected side (Enders and Seo, 2016). Suda et al. (2021) suggested that the light touch sensations of the thumb and the index finger were similar. Thus, different parts of the fingers have different receptors, thresholds, and types of sensations. Post-stroke sensory dysfunction is caused by a variety of factors, including damage to the focal brain areas that can affect hand function (Tyson et al., 2008; Umeki et al., 2018).

Somatosensory evoked potentials (SEP) are objective electrophysiological examinations that reflect proprioceptive and fine tactile conduction pathways, evaluate the sensory function, and predict prognosis. Compared to CT and MRI, SEP is less affected by aphasia, consciousness, and cognitive function and is more sensitive to stroke diagnosis (Sridharan et al., 2016; Macerollo et al., 2018). Feng et al. (2018) showed that more than half of patients with stroke had tactile abnormalities, which may be related to the loss or reduction of the higher central processing of sensory signals and changes in the somatosensory network of the brain in patients with stroke. Different waveforms of SEP represent different sources of neurogenesis, and N20 is one of the indexes that originates from the primary somatosensory cortex, which processes proprioception and fine touch. N20 is an objective, accurate, and sensitive index to examine the sensory function of patients with stroke (Macerollo et al., 2018). The prolonged latency and decreased amplitude of N20 on the affected side compared to the healthy side after stroke suggest that demyelination and axonal damage, respectively, may occur in early patients with stroke (Keren et al., 1993; Song et al., 2016). In addition, there are few studies on convalescent patients, and the parameters remain controversial. SEP can be affected by different factors; although it may be used as an auxiliary diagnostic tool, it should be used in conjunction with the patient’s clinical manifestations to make an objective and comprehensive assessment (Horn and Tjepkema-Cloostermans, 2017).

The main objective of this study was to investigate the characteristics and significance of light touch sensation and 2-PD in different parts of the hand in patients with stroke based on SEP analysis. To investigate the correlation between sensation and hemiplegic hand function, we aimed to identify more appropriate hand sensory measurement points with clinical significance and provide guidance for clinical sensory assessment and treatment.

## 2. Materials and methods

### 2.1. Participants

Patients with stroke treated in the third ward of the Department of Rehabilitation Medicine, Shijiazhuang People’s Hospital from June 2021 to August 2022, who met the diagnostic criteria for cerebrovascular disease, were selected for the study.

The inclusion criteria were as follows: (1) an age of 30–75 years; (2) first stroke with unilateral hemispheric lesions, including cerebral hemorrhage and infarction [criteria of the revised 4th National Cerebrovascular Disease Conference, 1995 (Neuroscience, and Surgery, 1997)]; (3) a disease course of 2 weeks–6 months; (4) sensory dysfunction defined as a degree of light touch sensation below grade 6 according to the Semmes–Weinstein Monofilaments (SWMs) test (Tyson et al., 2008) of at least one part of the hand, having a shortest 2-PD of > 5 mm for at least one part of the hand, or an abnormal SEP; (5) an ability to complete examinations; and (6) right-handedness evaluated by Edinburg handedness scale (Oldfield, 1971).

The exclusion criteria were as follows: (1) history of peripheral nerve damage, such as cervical spondylosis, trauma to the hands or upper limbs, and diabetic peripheral neuropathy; (2) Mini-Mental State Examination score of < 26, hemi-spatial neglect, body agony, mental, visual, hearing, and speech impairment; (3) sensory and motor dysfunction caused by other reasons; and (4) long-term use of drugs that provide nutrition for nerve or affect sensory function.

Sample size calculation. The formula for the sample size was *N* = $\frac{z_{1-\alpha /2}^{2}\,\left(1-p\right)}{\varepsilon^{2}\,p}$, where*z*_1−α/2_ is the percentage corresponding to an area of 1-α/2 under the standard normal distribution; *p* is the expected incidence rate; and ε is the percentage of the expected incidence rate. Using α = 0.05, ε= 0.1, *p* = 0.9 [according to the incidence of sensory dysfunction (Carlsson et al., 2018a), and realistic clinical situations], and*z*_1−α/2_ = 1.96, we calculated *N* = 43. Because this study used whole-group sampling, the actual sample size was multiplied by the design efficiency value, which was assumed to be 2 for *N* = 43. Thus, 86 cases were required; this study included 112 patients.

### 2.2. Procedure

The characteristics of the patients with stroke were recorded, including sex, age, physical labor intensity (Zhongliang, 1995), hemiplegic side, stroke type and location, disease course, Visual Analog Score (VAS), and Brunnstrom stage of the upper limbs and hands. Brunnstrom stage of motor function is as follows: flaccid without voluntary movement, stage I; causes joint movement of voluntary muscle contraction with the presence of spasticity, stage II; voluntary control of the movement with an intensified spasticity, stage III; spasticity starts to decline with more selective activation of muscles, stage IV; spasticity gradually recovers with a fine movement, stage V; presents individual joint movements and well-coordinated movement with normal muscle tone, stage VI (Brunnstrom, 1966). In addition, the following assessments were performed.

#### 2.2.1. Nerve electrophysiology

The test required a quiet room at 25^°^C and the patient’s skin at 32–34^°^C. The patient was quiet and had a clean skin surface. A Dandy Keypoint 4 EMG-evoked potential device (Denmark Alpine bioMed ApS Company) was used for testing. Median nerve stimulation of SEP involved placing the stimulating electrode 2–3 cm above the transverse wrist, using square wave stimulation with a duration of 0.2 ms, a frequency of 5 Hz, and slight thumb movement. Two SEP examinations were performed for each lateral cortical recording, each stacked 100 times. The placement of the recording electrodes was marked using the EEG International 10–20 system. The contralateral C3′ and C4′ were taken as recording points, and the reference electrode was placed at the FPz point and ipsilateral earlobe. After all electrodes are installed, resistance testing is performed. SEP can be recorded only when the electrode recording impedance is between 1 and 5 kΩ. The computer superimposed the signals generated by each stimulus in the center and traced the individual potentials. The N20 latency and wave amplitude were recorded. N20 waveforms without good repeatability, prolonged N20 latency, or decreased N20 amplitude were regarded as abnormal.

#### 2.2.2. Sensory function assessment

##### 2.2.2.1. Semmes–Weinstein monofilament testing

Semmes–Weinstein monofilaments testing (SMWT) were selected for light-touch sensory testing (Touch Test^®^ Complete Hand Kit, North Coast Medical Inc., USA). SWMT was performed with a constant length and different diameters of six light levels of nylon monofilaments: 0.07, 0.2, 2, 4, 10, and 300 g, representing grades 6, 5, 4, 3, 2, and 1, respectively. If the heaviest monofilament could not be measured by touch, a score of 0 was recorded. The test was performed with the patient’s eyes closed. Starting with the smallest value, the patient informed the examiner immediately when they felt a touch sensation. When using the 0.07 and 0.2 g wires, each wire was used three times by applying the wire to the skin for 1–1.5 s and then lifting it for 1–1.5 s. Each filament from 2 to 300 g was applied once only. When the nylon monofilament was bent, and the patient was still unable to feel it, the wire was changed to a heavier wire and retested. If the patient felt it two times in a row, the researcher recorded the number for the result. Five measurement sites innervated by the median and ulnar nerves were assessed, including the palmar aspect of the thumb, the index finger, the little finger, thenar, and hypothenar (Fedorczyk, 2002). The interval for each measurement site was at least 5 s. Grade 6 was considered sensory intactness; grades 2–5 were considered sensory impairment; and grades 0–1 were considered a sensory loss. Whereby at least two of the five sites on the affected side were considered to have a poor light touch sensation if the result was = 2. In addition, if one or none of the five sites got a grade of = 2, we considered it to have a good light touch sensation (Tyson et al., 2008; Peng et al., 2018). The thumb, the index finger, and the little finger were classified as the finger group. The thenar and hypothenar were classified as the palm group. The same is as the following 2-PD.

##### 2.2.2.2. 2-PD

The 2-PD test tool (Touch Test^®^ Complete Hand Kit, Two-Point Discriminator, North Coast Medical Inc., USA) was used to evaluate 2-PD. With the patient’s eyes closed, the examiner recorded the smallest distance that could be felt between two points. The measurement range was 2–15 mm, and measurements > 15 mm were out of range. The test site was the same as that used for light touch sensation testing. The interval for each measurement site was at least 5 s. Patients’ fingers with a 2-PD of 2–5, 6–15, and > 15 mm were considered to have sensory intactness, sensory impairment, and sensory loss, respectively (Peng et al., 2018). The standard of the patients’ palms was that < 12 mm was regarded as sensory intactness, 12–15 mm was regarded as sensory impairment, and > 15 mm was regarded as sensory loss (Dawei et al., 2018). We defined > 15 mm as 16 mm. If two of the five sites’ results on the affected side were > 15 mm, the hand was considered to have lost 2-PD (Tyson et al., 2008; Peng et al., 2018).

##### 2.2.2.3. Motor function assessment

Hemiplegic hand function was used to assess the hand function of patients with stroke. Patients were required to gradually complete five kinds of movements. If they could not complete all the tasks, the grade was recorded as I. If they could complete 1, 2, 3, 4, or 5 kinds of movement, then the grades were recorded as II, III, IV, V, and VI, respectively (Zhao et al., 2022).

### 2.3. Data analysis

Statistical software (SPSS 26.0; IBM, Chicago, Illinois, USA) was used to analyze the data. Measurement data that conformed to a normal distribution are expressed as ($\bar{x}$± SD), while data that did not conform to a normal distribution are expressed as median (*P25–P75*). Differences were considered statistically significant at *p* < 0.05. The light touch impairment level of the thumb, the index finger, and the little finger was aggregated for each patient as the impairment level for the finger group. The final distribution of light touch sensation in the finger group was obtained by summing all included patients. The distribution of light touch sensation in the palm group was obtained by summing the impairment grades of the thenar and hypothenar for all patients. 2-PD was calculated in the same way. The Pearson correlation required the two sample data distributions to be normal. If there were variables that did not conform to a normal distribution, Spearman’s correlation was used. We make the correlation between the grade of light touch sensation and N20 parameters. The correlation between the distance of 2-PD and N20 parameters was also made. Wilcoxon signed-ranks tests were used to compare the light touch sensation, 2-PD, and N20 amplitude and latency between the unaffected and affected sides of patients with stroke. The mean levels of light touch sensation and 2-PD in the fingers (including the thumb, the index finger, and the little finger) and palm (including the thenar and hypothenar) were counted separately, and the percentage of their corresponding numbers was calculated. Within- and between-group comparisons were performed using the Mann–Whitney, Kruskal–Wallis, and chi-square tests, and multiple comparisons were performed using the S-N-K method if the data distribution locations were not the same between groups. The Spearman correlation was performed between the light touch sensation and the N20 wave amplitude, latency, and hemiplegic hand function for all testing sites in the included patients. 2-PD was performed with the same Spearman correlation as the light touch sensation.

## 3. Results

**FIGURE 1:**
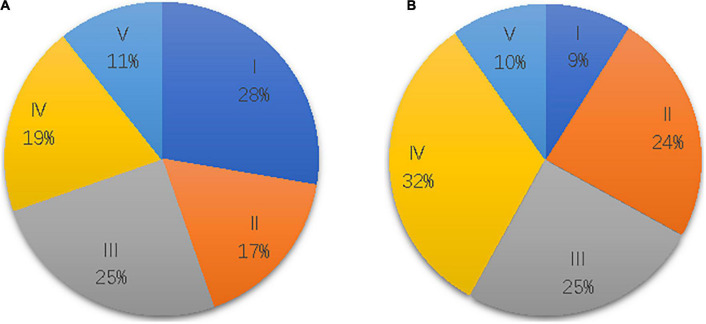
Brunnstrom stage distributions in the hand **(A)** and upper limb **(B)** of 112 patients.

A total of 112 patients with stroke, age (54.13 ± 11.87) years, were included in this trial. There were 48 cases of cerebral hemorrhage and 64 cases of cerebral infarction; 79 patients were male and 33 were female. All participants were right-handed before the stroke. Detailed clinical patient characteristics are presented in Table 1, and the Brunnstrom stage data are presented in Figure 1.

**TABLE 1:** Patients’ characteristics.

Characteristics	
**Sex, *n***	
Female	79
Male	33
Age, years, mean ± SD	54.13 ± 11.87
**Physical labor intensity, *n***	
Light physical labor	63
Moderate physical labor	35
Heavy physical labor	14
**Hemiplegic side, *n***	
Left	59
Right	53
**Type of stroke, *n***	
Ischemic	48
Hemorrhagic	64
**Location**	
Cortical	6
Subcortical	71
Mixture of cortical and subcortical lesions	35
Course of the stroke, days, median (*P*_25_–*P*_75_)	30 (19–60)
VAS, median (*P*_25_–*P*_75_)	0 (0–2)
Fugl-Meyer score, median (*P*_25_–*P*_75_)	22 (8, 46)
**Brunnstrom stage, median (*P*_25_–*P*_75_)**	
Hand	3 (1–4)
Upper extremities	3 (2–4)

SD, standard deviation; VAS, visual analog scale.

### 3.1. Comparison of light touch sensation and 2-PD in patients with stroke

**FIGURE 2:**
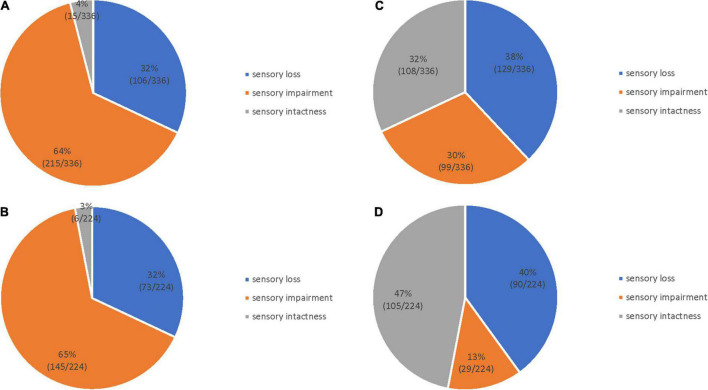
Sensory dysfunction distribution in 112 patients with stroke. Light touch sensation in the finger **(A)** and palm **(B)** regions; 2-PD in the finger **(C)** and palm **(D)** regions.

The distribution of light touch sensation and 2-PD in the finger and palm regions of patients with stroke is shown in Figure 2. Figures 2A, B show the level of light touch sensation in the finger and palm regions, respectively. The light touch sensation level was dominated by sensory impairments in both the finger and palm regions. The 2-PD level of the finger region was dominated by sensory loss (Figure 2C), and the palm region was mainly sensory intactness (Figure 2D). The degree of 2-PD dysfunction was worse in the palm region than in the finger region. There were significant differences in the levels of light touch sensation and 2-PD impairment in both finger and palm regions (Table 2).

**TABLE 2:** The level of sensory in the finger and the palm region.

		Finger region	Palm region
The level of light touch sensation	Sensory loss	106	73
	Sensory impairment	215	145
	Sensory intactness	15	6
	*z*	0.404	0.407
	*p*-value	<0.001	<0.001
The level of 2-PD	Sensory loss	129	90
	Sensory impairment	99	29
	Sensory intactness	108	105
	z	0.279	0.385
	*p*-value	<0.001	<0.001

### 3.2. Distribution of patients with stroke and the corresponding degree of light touch sensation dysfunction in five hand regions

There was no significant difference in the number of patients corresponding to the degree of sensory dysfunction among the various parts of the fingers (*x*^2^ = 1.344, *p* = 0.511), and the distribution of the degrees of sensory intactness, impairment, and loss in the different locations was not significant (*x*^2^ = 0.556, *p* = 0.757) (Table 3).

**TABLE 3:** Degree of light touch sensation dysfunction.

	Thumb	Index finger	Little finger	Thenar	Hypothenar	x^2^	*P*-value
Sensory loss	33	36	37	36	37	0.556	0.757
Sensory impairment	76	67	72	72	73		
Sensory intactness	3	9	3	4	2		
**x^2^**	1.344						
*p*-value	0.511						

### 3.3. Distribution of participants corresponding to the degree of 2-PD dysfunction in five hand regions

There was a statistically significant difference in 2-PD among the five parts. Multiple comparisons showed a statistically significant difference among the thenar and thumb, the index finger, and the little finger (*p* < 0.001). There was also a statistically significant difference between the hypothenar and thumb, the index finger, and the little finger (*p* < 0.001). However, there was no statistical difference between the fingers (the thumb, the index finger, and the little finger) or between the thenar and hypothenar regions.

The three degrees of 2-PD impairment were statistically different at different locations (*p* = 0.525), with most 2-PD graded as sensory loss (39.1%) (Table 4).

**TABLE 4:** Degree of 2-PD dysfunction.

	Thumb	Index finger	Little finger	Thenar	Hypothenar	x^2^	*P*-value
Sensory loss	43	42	44	45	45	3.200	0.525
Sensory impairment	34	31	34	13	16		
Sensory intactness	35	39	34	54	51		
**x^2^**	16.658						
*p*-value	<0.001						

2-PD, two-point discrimination.

### 3.4. Correlation of light touch sensation and 2-PD

The two sides of the patients with stroke had significant differences in light touch sensation and 2-PD (*p* < 0.001). In a total of 112 patients, 44 patients had good light touch sensation and 36 patients had a 2-PD sensory loss. There were 8 patients without good light touch sensation and having 2-PD sensory.

### 3.5. Correlation of SEP with light touch sensation and 2-PD

The included patients had an elicitation rate of 93.75%; seven patients did not elicit N20 waveforms with good repeatability. Of the included patients, 65 had a decreased N20 amplitude and/or prolonged latency, with an abnormal rate of 61.32%. The two sides of the patients with stroke demonstrated a significant difference in the N20 latency and amplitude (latency: z = −3.752, *p* < 0.001; amplitude: z = −4.428, *p* < 0.001). The N20 amplitude was positively correlated with 2-PD and light touch sensation in each part of the hand, and all were statistically different. The correlation coefficient between the N20 amplitude and 2-PD in the little finger was the largest (*r* = −0.409, *p* < 0.001) (Table 5).

**TABLE 5:** The relationship between sensation and N20.

	N20 amplitude				N20 latency			
	Light touch sensation		2-PD		Light touch sensation		2-PD	
	*r*	*P*-value	*r*	*P*-value	*r*	*P*-value	*r*	*P*-value
Thumb	0.243	0.012	−0.368	<0.001	−0.105	0.288	−0.107	0.278
Index finger	0.276	0.004	−0.338	<0.001	−0.103	0.297	−0.127	0.196
Little finger	0.253	0.009	−0.409	<0.001	−0.158	0.107	−0.158	0.107
Thenar	0.279	0.004	−0.277	0.004	−0.147	0.134	−0.153	0.118
Hypothenar	0.257	0.008	−0.315	<0.001	−0.136	0.166	−0.136	0.166

2-PD, two-point discrimination; N20, negative peak at 20 ms.

### 3.6. Correlation of hemiplegic hand function with sensation

The grade of hemiplegic hand function in patients with stroke was positively correlated with the light touch sensation of each part of the affected side and negatively correlated with 2-PD. The correlation coefficient between the 2-PD of the little finger and hemiplegic hand function (*r* = −0.359, *p* < 0.001) was the largest (Table 6).

**TABLE 6:** The relationship between hemiplegic hand function and sensation.

	Light tough sensation		2-PD	
	*r*	*P*-value	*r*	*P*-value
Thumb	0.272	0.004	−0.342	<0.001
Index finger	0.259	0.006	−0.340	<0.001
Little finger	0.258	0.006	−0.359	<0.001
Thenar	0.222	0.019	−0.245	0.011
Hypothenar	0.251	0.008	−0.235	0.015

2-PD, two-point discrimination.

## 4. Discussion

Post-stroke sensory dysfunction not only affects patients’ perception of pins, needles, temperature, and pain (Goodin et al., 2018) but also affects motor function and activities of daily living (Carlsson et al., 2018a). The current clinical procedures for sensory assessment are complex, and the criteria are unclear; therefore, more comprehensive and accurate assessments are needed to provide a basis for training. This study evaluated 112 patients with stroke with at least one type of sensory dysfunction and found that light touch sensation was dominated by sensory impairment, and the distribution of light touch sensation was different in both the finger and palm regions, with no significant difference. Both finger and palm regions experienced sensory impairment (65%), and sensory intactness was the least experienced. One study using the same assessment method and criteria calculated a rate of light touch sensory loss of approximately 25.5% (Tyson et al., 2008), compared to 32% in this study. This may be because our study included convalescent stroke patients with sensory dysfunction but did not include patients with stroke with normal sensations (Tyson et al., 2008). The 2-PD distribution in the palm and finger regions was different; the finger region experienced predominantly sensory loss (38%) and the palm region experienced predominantly sensory loss (39%). The hands have fine motor skills, and the sensory cortex occupies a large proportion of the functional areas of the brain (Williams and Warwick, 1973). Hand sensory impairment is one of the common manifestations of hemiplegia, which affects the flexibility of hand movements (Kitai et al., 2021). We found that the impairment of 2-PD was worse than that of light touch sensation in patients with stroke, which is consistent with Lima et al. (2015) and Wolny et al. (2017). However, Tyson et al. (2008) found no significant difference in light touch sensation and 2-PD among the elbow, the wrist, and the thumb. This may be because they included patients with stroke at 2–4 weeks who were more severe and had poorer motor functions. In addition, anxiety, depression, and cognitive impairment affect patient perceptions (Paolucci, 2017).

Light touch sensation and 2-PD differed in patients with stroke at different measurement points, and the site of choice may reflect different functional levels. Peng et al. (2018) found that 2-PD in the index finger was negatively correlated with the Fugl-Meyer Assessment of the upper extremities (FMA-UE) in elderly patients with stroke, whereas 2-PD in the thenar was not correlated with FMA-UE. There was a significant difference in sensory function between the unaffected and affected sides of the thumbs and the index fingers in patients with stroke but not in the thenars (Peng et al., 2018). Enders and Seo (2016) found that stroke patients with sensory impairment had greater deviations from the normal strength measured in each finger than those without sensory impairment, suggesting that differences in the pinch grip control in each finger may be related to hyperalgesia in each finger. Similar results were found in the present study; there was no significant difference in light touch sensation among all parts of the hand, and there was a significant difference in 2-PD among all parts of the hand, providing a basis for clinical evaluations. Carlsson et al. (2018b) studied the effect of sensorimotor training in patients with stroke and more thoroughly examined the effect of treatment by performing a sensory analysis of digits I, II, and V in the thenar and hypothenar regions. Although such an evaluation method improves the accuracy of the objective examination, it is time-consuming. Whether this represents the true nature of sensory impairment is worth investigating.

The SEP, a neurophysiological detection method commonly used in clinical practice, is stimulated mainly by the Ia fiber at the end of the limb, reflecting the conductive proprioceptive and fine tactile sensations, such as 2-PD and the tactile sensation of discriminating the texture of objects, of the posterior cord medial tegmental system. Currently, it is the most objective method for evaluating sensory pathways (Karnaze et al., 1987). The waveforms of the SEP latency could indicate the source of neurogenesis; N20 is generally considered to represent the cortical activity of the postcentral gyrus recorded during the stimulation of the median nerve (Tamura et al., 2009). We found that the N20 amplitude was mostly reduced and correlated with the level of sensory function in patients with stroke. Some studies found that early patients with stroke had predominantly axonal damage, manifested by a decreased N20 amplitude. The reason why the N20 latency was not correlated with the sensory function was that there were fewer stroke patients with demyelinating changes (Wang et al., 2012; Chen et al., 2021; Yoon et al., 2021). Al-Rawi et al. (2009) showed that the N20 amplitude correlated with the Medical Research Council score and could represent the level of function in patients with stroke, which was consistent with the results of this study. We found that light touch sensation and 2-PD had no relationship with the N20 latency. Tzvetanov and Rousseff (2005) found no correlation between the N20 latency and limb function in patients with acute stroke, which is consistent with our results. Keren et al. (1993) found that the median nerve SEP with a prolonged latency and shortened amplitude was also associated with poor motor function. It may be related to the fact that the SEP component studied by Keren et al. (1993) was not N20. Few studies have examined the correlation between SEP and sensory function in patients with stroke. The variations in these studies may be related to the duration of the disease, age, and other factors.

In this study, we found that both light touch sensation and 2-PD were correlated with the N20 amplitude. Light touch sensation was not significantly different among all parts of the hand and was weakly correlated with the N20 amplitude. The correlation coefficients between the light touch sensation of the hand and the N20 amplitude were different, with smaller correlation coefficients for the palm of the hand (thenar and hypothenar) and larger correlation coefficients for the fingers (with the little finger > thumb > index finger). Therefore, it is recommended that when screening the sensation of patients with stroke, any part of the fingers or palm should be selected to assess light touch sensation. For 2-PD, the finger and palm regions represented different sensory states; we could select the little finger for the fingers and any part of the palm. Suda et al. (2021) found that the light touch sensation between the thumb and the index finger was not significant and was consistent with the results of our study. In contrast, 2-PD is a complex sensation that requires not only a complete medial thalamus sensory transmission pathway but also a deficit in cognitive function that could affect the discrimination of general sensations during the assessments (Harvey, 2019). It was reported that there was a correlation between sensory and cognitive function in a normal aging sample (Tay et al., 2006). Lin and Jia (2020) demonstrated that cognitive function recovery is important in the areas of sensory, memory, attention, and emotion. Sensory training is inseparable from good attention and thinking and memory skills and influences cognitive behaviors such as attention and perception (Lin and Jia, 2020). The accuracy and authenticity remain to be verified.

Most studies have shown that sensory and motor functions are closely related to patients with stroke. Meyer et al. (2014) performed a meta-analysis of six articles to examine the relationship between proprioceptive deficits and function in patients with stroke and found that light touch sensation combined with proprioception was significantly associated with upper limb motor function and activities of daily living. To study the effect of intensive sensorimotor training in patients with stroke, Diego et al. (2013) divided patients into two groups: a control group undergoing conventional rehabilitation and an experimental group undergoing additional intensive sensorimotor training. The results showed that the latter was more statistically significant in the Fugl-Meyer test. The results of our study showed that the hemiplegic hand function was positively correlated with the light touch sensation of the hand and negatively correlated with 2-PD, which is more consistent with the results of Diego et al. (2013) and Meyer et al. (2014). Sensory function decreases after stroke and correlates with hemiplegic hand function, with the highest correlation coefficient observed in the thumb. To improve disabilities in patients with stroke, more sensory stimulation of the thumb can be provided. In addition, this study also found 10 patients with a normal light touch sensation but with 2-PD loss, 35 patients presenting with both light touch sensation and 2-PD loss, and only 1 patient presenting with a light touch sensation with normal 2-PD. This was generally consistent with the majority of studies that demonstrate that light touch sensation could be exempted from the 2-PD test if it was lost (Barnett, 1998; Carlsson et al., 2018a). However, our study also found that light touch sensation was poor, whereas 2-PD may not be poor, and 2-PD is related to the ability to perform daily activities. Therefore, assessing 2-PD in the sensory assessment was necessary if the light touch sensation was poor. The little finger and hypothenar may also better represent the function of 2-PD.

However, this study had some limitations. First, the difference between two adjacent monofilaments is not very large so that it may lead to the limited use of SWMT. Furthermore, light touch sensation and 2-PD may not be simple examinations of superficial and compound sensory sensations. The formation of light touch sensation and 2-PD was complex, and the assessment process was influenced by the patient’s injury location and subjective consciousness, which may influence the accuracy. The education level, occupation, living habits, etc., may be factors that affect the epidermal thickness, which may affect sensation (Zimmerman et al., 2014; Fu et al., 2016). It is worth exploring whether they represent superficial and compound sensory stimuli. In this study, when the patient could not feel the touch of the heaviest monofilament, they got a score of 0. However, for example, patients may feel a monofilament weighing 448 g, a score of 0 was still credited according to the criteria of this study. It may confuse the level of this patient’s light touch sensation. The weight span of the monofilament selected in this study is large, which limits its use in the evaluation. Second, SEP is a highly sensitive neurophysiological index that may be influenced by noise as well as physiological factors such as height and weight. In addition, the disease duration may be a factor that influences N20, which may explain the lack of a statistical difference between the sensory function and N20 latency. Besides that, we just exclude the patients with a history of cervical spondylosis, which had an effect on their daily living through their CT or MRI of the cervical spine. Those patients who did not undergo a relative examination presenting mild symptoms were not completely excluded. Furthermore, tremors or spasms are a source of noise in patients after stroke, which in turn affects SEP. The sweep number of SEP was only 100 accumulation in this study. In the next study, we will consider the noise of SEP and expand the sweep number of SEP to 500 accumulate (Tashiro et al., 2019). Therefore, further research will involve examining shorter disease durations. Finally, this study only evaluated light touch sensation and 2-PD without screening for deep sensation; therefore, more in-depth and comprehensive studies are needed on the dysfunction of sensory function in patients with stroke.

## Data availability statement

The original contributions presented in this study are included in the article/supplementary material, further inquiries can be directed to the corresponding author.

## Ethics statement

The studies involving human participants were reviewed and approved by the North China University of Science and Technology. The patients/participants provided their written informed consent to participate in this study.

## Author contributions

YL: project conception, writing the original manuscript, data analysis, and investigation. JM: project administration, methodology, and modifying the manuscript. HL: data curation and review and editing the manuscript. W-YS: patients’ interview and methodology. Z-HX: data analysis and investigation. QY: evaluation of the patients and data analysis. Q-QZ: evaluation of the patients. FW: data analysis. X-LT: patients’ interview. Y-FB: quality control. All authors contributed to the article and approved the submitted version.
